# Ginsenoside Rg1 Ameliorates Rat Myocardial Ischemia-Reperfusion Injury by Modulating Energy Metabolism Pathways

**DOI:** 10.3389/fphys.2018.00078

**Published:** 2018-02-07

**Authors:** Lin Li, Chun-Shui Pan, Li Yan, Yuan-Chen Cui, Yu-Ying Liu, Hong-Na Mu, Ke He, Bai-He Hu, Xin Chang, Kai Sun, Jing-Yu Fan, Li Huang, Jing-Yan Han

**Affiliations:** ^1^Department of Integrative Cardiology, Beijing China-Japan Friendship Hospital, Beijing, China; ^2^Tasly Microcirculation Research Center, Peking University Health Science Center, Beijing, China; ^3^Key Laboratory of Microcirculation, State Administration of Traditional Chinese Medicine of the People's Republic of China, Beijing, China; ^4^Key Laboratory of Stasis and Phlegm, State Administration of Traditional Chinese Medicine of the People's Republic of China, Beijing, China; ^5^Beijing Microvascular Institute of Integration of Chinese and Western Medicine, Beijing, China; ^6^State Key Laboratory of Core Technology in Innovative Chinese Medicine, Beijing, China; ^7^Department of Integration of Chinese and Western Medicine, School of Basic Medical Sciences, Peking University, Beijing, China

**Keywords:** panax notoginseng, cardiac function, ATP synthase subunit, RhoA, energy metabolism

## Abstract

As a major ingredient of *Radix ginseng*, ginsenoside Rg1 (Rg1) has been increasingly recognized to benefit the heart condition, however, the rationale behind the role is not fully understood. *In vitro* study in H9c2 cardiomyocytes has shown the potential of Rg1 to increase ATP content in the cells. We thus speculated that the protective effect of Rg1 on heart ischemia and reperfusion (I/R) injury implicates energy metabolism regulation. The present study was designed to verify this speculation. Male Sprague-Dawley rats were subjected to 30 min of occlusion of left coronary anterior descending artery followed by reperfusion for 90 min. Rg1 (5 mg/kg/h) was continuously administrated intravenously 30 min before occlusion until the end of reperfusion. Myocradial blood flow and heart function were monitored over the period of I/R. Myocardial infarct size, structure and apoptosis, energy metabolism, and change in RhoA signaling pathway were evaluated 90 min after reperfusion. Binding of Rg1 to RhoA was assessed using Surface Plasmon Resonance (SPR). Rg1 prevented I/R-elicited insults in myocardium, including myocardial infarction and apoptosis, decreased myocardial blood flow (MBF) and heart function, and alteration in myocardium structure. Rg1 restored the production of ATP in myocardium after I/R. Rg1 was able to bind to RhoA and down-regulate the activity of RhoA signaling pathway. These results indicated that Rg1 had protective potential against I/R-induced myocardial injury, which may be related to inhibiting myocardial apoptosis and modulating energy metabolism through binding to RhoA.

## Introduction

Ischemic heart disease (IHD) is the leading cause of death and disability worldwide. Until now, the most effective strategy for IHD is timely percutaneous coronary intervention (PCI). However, the PCI itself can induce myocardial reperfusion injury (Braunwald and Kloner, 1985; Yellon and Hausenloy, 2007).

Ischemia and reperfusion (I/R) injury is a pathogenesis process consisting of a spectrum of episodes, among which mitochondria dysfunction plays a central role. Mitochondria as an energy station in the cell provide the majority of ATP using oxygen and nutrition, which is obligatory for the cell activity and essential for keeping cell structure integrity as well. Ischemia halts oxidative phosphorylation in mitochondria, decreases the activity, and/or impairs the expression of respiratory chain complexes (He et al., 2014; Mu et al., 2015; Yang et al., 2015), leading to decrease in ATP synthesis and ATP deprivation (Burwell et al., 2009; Wheaton and Chandel, 2011; Mu et al., 2015; Yang et al., 2015). ATP depletion causes depolymerization of F-actin and disarrangement of thin filament of myocardial cells (Korn et al., 1987), impairs the interaction between myocardial thin filament and thick filament and subsequent myosin cross-bridge cycle (Han and Ogut, 2010), results in abnormality of cardiac structure and contractile function ultimately. Mitochondria are also the major source of reactive oxygen species (ROS), particularly on reperfusion when a surge of oxygen overflows the respiratory chain, where the electrons accumulated in the impaired complex I and II may escape and combine with oxygen to form peroxides. In addition, mitochondria malfunction initiates apoptosis, which, along with ROS, exaggerates the energy depletion-caused myocardial injury (Pell et al., 2016; Han et al., 2017). Thus, protection of mitochondria and amelioration of energy deficiency during myocardial I/R are crucial in IHD therapy. However, the regime for this purpose is limited at present.

Cardiac I/R is characterized by complex alterations in fatty acid and glucose metabolism. Fatty acid oxidation is limited by the lack of oxygen in ischemia, accompanying with an accelerated anaerobic glycolysis. When oxygen delivery is restored during reperfusion, the residual AMPK accelerates fatty acid oxidation (Qi and Young, 2015). Pharmacologically shifting the balance between fatty acid β-oxidation and glycolysis can improve the efficiency of ATP generation (Lopaschuk and Stanley, 2006; Frank et al., 2012), and reduce I/R injury.

Ginsenoside Rg1 (Rg1, the molecule structure is shown in **Figure 9A**) is an active component derived from herbal medicine *Radix ginseng* (Renshen), one of the major medicines to treat Qi-deficiency related diseases in traditional Chinese medicine. Recent pharmacological studies found that Rg1 is able to suppress myocardial infarction area (Wang et al., 2010), enhance the myocardial perfusion and preserve left ventricle (LV) function (Wei et al., 2007; Yin et al., 2011), as well as ameliorate ventricular remodeling in acute or chronic myocardial infarction animal model (Yin et al., 2011). Furthermore, *in vitro* experiment suggested that Rg1 protects rat cardiomyocyte from hypoxia/reoxygenation (H/R) oxidative injury via regulation of antioxidant and intracellular calcium homeostasis (Zhu et al., 2009). Rg1 was reported to inhibit autophagosomal formation and apoptosis in H/R-induced H9c2 cardiomyocytes, which was associated with the increase of cellular ATP content and the relief of oxidative stress (Zhang et al., 2012; Li et al., 2017). However, few *in vivo* study is available with respect to the protective role of Rg1 in I/R-induced myocardial injury, particularly the underlying mechanism. We hypothesized that Rg1 may protect myocardium from I/R injury through modulating energy metabolism. The present study was aimed to verify this hypothesis.

## Materials and methods

### Animals

Male Sprague-Dawley rats (240–260 g) were obtained from the Animal Center of Peking University Health Science Center with the certificate number SCXK 2006-0008, which were raised with standard diet at temperature 22 ± 2°C and humidity 40 ± 5% under a 12-h light/dark cycle. The animals were fasted for 12 h before the experiment while allowing access to water freely. The experimental procedures were in accordance with the recommendations of UK. Animals (Scientific Procedures) Act, 1986 and associated guidelines, EU Directive 2010/63/EU for animal experiments. All animals were handled according to the guidelines of the Peking University Animal Research Committee. The experimental protocol was approved by the Committee on the Ethics of Animal Experiments of Peking University Health Science Center (LA2016314).

### Drug and reagents

Rg1 was obtained from Feng Shan Jian Medicine Research Co. Ltd. (Kunming, China). Evans blue and 2,3,5-triphenyltetrazolium chloride (TTC) were purchased from Sigma (St. Louis, MO, USA). ELISA kits for ATP, ADP, AMP, and cTnI were from Beijing Andihuatai Technology Co. Ltd. (Beijing, China). The enzyme activity assay kits of complex I, complex II, and ATP synthase, the primary antibodies against cTnI, ROCK1, phospho-RhoA, RhoA, phospho-MYPT1, human RhoA full-length protein, and ATP5D were obtained from Abcam (Cambridge, MA, USA). The primary antibodies against Bax, Bcl-2, cleaved-caspase-3, MYPT1, and GAPDH were obtained from Cell Signaling Technology (Beverly, MA, USA).

### Experimental protocol

SD rats were randomly divided into four groups: Sham, Rg1+Sham, I/R, and Rg1+I/R. Myocardial I/R model was established by ligation of left coronary anterior descending artery for 30 min followed by 90 min reperfusion, as previously described (Lin et al., 2013). The animals in Sham and Rg1+Sham group underwent the same procedure but without ligation. In Rg1+Sham and Rg1+I/R group, the animals received a continuous infusion of Rg1 (5 mg/kg/h) (Sun et al., 2007) via a femoral vein catheter starting from 30 min before ischemia until the end of reperfusion (totally 150 min). The animals in Sham and I/R group received continuous intravenous infusion of normal saline over the same period of time.

### Myocardial infarct size determination

LADCA was ligated at 90 min after I/R, and 2 mL of 0.35% Evans blue was administrated through femoral vein. Hearts were rapidly excised and cut into five slices of 1 mm thick, parallel to the atrioventricular groove from the apex cordis to the ligation site. Slices were incubated for 15 min at 37°C in a 0.375% solution of TTC, and photographed with a stereomicroscope connected with Digital Sight (DS-5M-U; Nikon, Nanjing, China). In so treated sections, the degree of myocardial tissue injury was discriminated by different colors with infarction zone (IA) being stained white, area at risk (AAR) pink, and non-IA blue. The myocardial areas of infarct, AAR, and LV were analyzed by Image-Pro Plus 6.0 (Media Cybernetic, Bethesda, MD, USA) (Lin et al., 2013).

### Myocardial blood flow estimation

The myocardial blood flow (MBF) was measured by Laser-Doppler Perfusion Imager (PeriScan PIM3 System; PERIMED, Stockholm, Sweden) equipped with a computer at baseline, immediately after ischemia, and 30, 60, and 90 min after reperfusion. A color-coded image was displayed on a video monitor, and all images were evaluated with the software LDPIwin 3.1 (PeriScan PIM3 System; PERIMED, Stockholm, Sweden). The magnitude of MBF was represented by different colors, with blue to red denoting low to high. Results were expressed as percentages of the baseline.

### Heart function assessment

A cannulation was inserted into LV through right carotid artery, which was connected to a bio-function experiment system BL-420F (Chengdu Taimen Technology Ltd., Chengdu, China). The heart function was evaluated at baseline, immediately after ischemia, and 30, 60, and 90 min after I/R as described previously (Lin et al., 2013).

### Ultrastructure examination

Rat hearts were perfused for 40 min with 4% paraformaldehyde and 2% glutaraldehyde (TedPella, Redding, CA, USA) in 0.1 mol/L phosphate buffer at a speed of 3 ml/min, after which the hearts were removed. Myocardial tissue was collected at one-third above the apex cordis from the surrounding infarct region of LV and cut into 1 mm^3^ blocks. The tissue blocks were fixed overnight at 4°C with 3% glutaraldehyde, washed three times with 0.1 mol/L phosphate-buffered solution, and then post-fixed with 1% osmium tetraoxide for 2 h. The ultrathin sections were prepared as routine, observed, and photographed with a transmission electron microscope (JEM 1400 plus, JEOL, Tokyo, Japan) (Lin et al., 2013).

### Determination of cTnI in serum

At 90 min after reperfusion, anticoagulant blood was collected, centrifuged (1,000 g, 4°C) for 15 min, and the supernatant was harvested. The serum contents of cTnI were determined using a rat cTnI ELISA Kit (Andihuatai Technology Co. Ltd., Beijing, China) and detected by microplate reader (MULTISKAN MK3; Thermo, San Jose, CA, USA).

### Confocal microscopy for fluorescence staining of F-actin and apoptotic cells

Double staining of rhodamine phalloidin and terminal-deoxynucleotidyl transferase mediated nick end labeling (TUNEL) for myocardial tissue sections was conducted at 90 min after reperfusion, as described previously(Tu et al., 2013). In brief, heart was removed and fixed in 4% paraformaldehyde solution for 48 h, processed for paraffin section (5 μm). TUNEL staining was undertaken using a cell death detection kit (Roche, Basel, Switzerland), followed by counterstaining with rhodamine phalloidin (Invitrogen, Carlsbad, CA, USA) for F-actin and Hoechst 33342 for nuclei. Five fields were selected from the surrounding of infarction areas of the LV for each section at 40X magnification of objective, and observed with a Laser Scanning Confocal Microscope (TCS SP5; Leica, Mannheim, Germany). The number of the TUNNEL-positive cells in the five fields was counted, averaged, and expressed as cell number per field.

### ELISA for energy metabolism status

Rat hearts were removed after 90 min I/R under anesthesia. The LV tissue sample was taken at 2 mm under ligature, frozen in liquid nitrogen, and stored at −80°C. The whole protein of the myocardial tissues was extracted with a protein extraction kit (Applygen Technologies, Beijing, China). The content of ATP, ADP, and AMP in myocardial tissue was assessed with ELISA kits (Andihuatai Technology Co. Ltd., Beijing, China) by microplate reader (MULTISKAN MK3, Thermo, San Jose, CA, USA) according to the manufacturer's instructions, as described previously (Tu et al., 2013). The activity of complex I, complex II and ATP synthase in myocardial tissue was assessed with ELISA kits (Andihuatai Technology Co. Ltd., Beijing, China) and detected by microplate reader (MULTISKAN MK3, Thermo, San Jose, CA, USA) according to the manufacturer's instructions, as described previously (He et al., 2014).

### Western blotting assay

Myocardial tissue was harvested 90 min after reperfusion from the lower and front part of LV, the only region that was revealed by Evens blue staining to have the ischemic tissue. The myocardial tissue was homogenized in lysis buffer containing the protease inhibitor. Equivalent amount of proteins were loaded on the membranes and incubated with antibodies against cTnI (1:2,000), phospho-RhoA (1:1,000), RhoA (1:1,000), Bax (1:1,000), Bcl-2 (1:1,000), cleaved-Caspase-3 (1:1,000), ROCK1 (1:1,000), MYPT1 (1:1,000), phosphor-MYPT1 (1:1,000), MLC (1:1,000), phosphor-MLC (1:1,000), ATP5D (1:1,000), GAPDH (1:2,000), respectively, followed by incubation with corresponding secondary antibodies. The GADPH western blotting was performed for each membrane as a loading control. Semi-quantitation of the protein was undertaken using Image-Pro Plus 5.0 software (Bethesda, MD, USA). The result of each group was expressed as a relative optical density to that from Sham group.

### Surface plasmon resonance (SPR)

Surface Plasmon Resonance (SPR) was used to evaluate the capacity of Rg1 to bind to RhoA. Carboxymethylated (CM5) sensor chip was docked into the T200 (Biacore, GE Healthcare, Sweden). Human RhoA full-length protein (Abcam, Cambridge, MA, USA) was immobilized on a CM5 sensor chip by injecting RhoA. Rg1 was injected from low to high concentration to eliminate artifacts in the data from adsorption carryover on the instrument flow. Equilibrium dissociation constant (KD) was calculated by fitting a 1:1 Langmuir model using Biacore T200 evaluation software v2.0 (Biacore, GE Healthcare, Sweden).

### Data analysis

All parameters were expressed as mean ± standard error (SE). Statistical analysis was performed using one-way ANOVA followed by Newman-Keuls test or two-way ANOVA (MBF and cardiac function) followed by Bonferroni for multiple comparisons. A *p* < 0.05 was considered to be statistically significant.

## Results

### Rg1 diminishes myocardial infarct size induced by I/R

**Figure 1:**
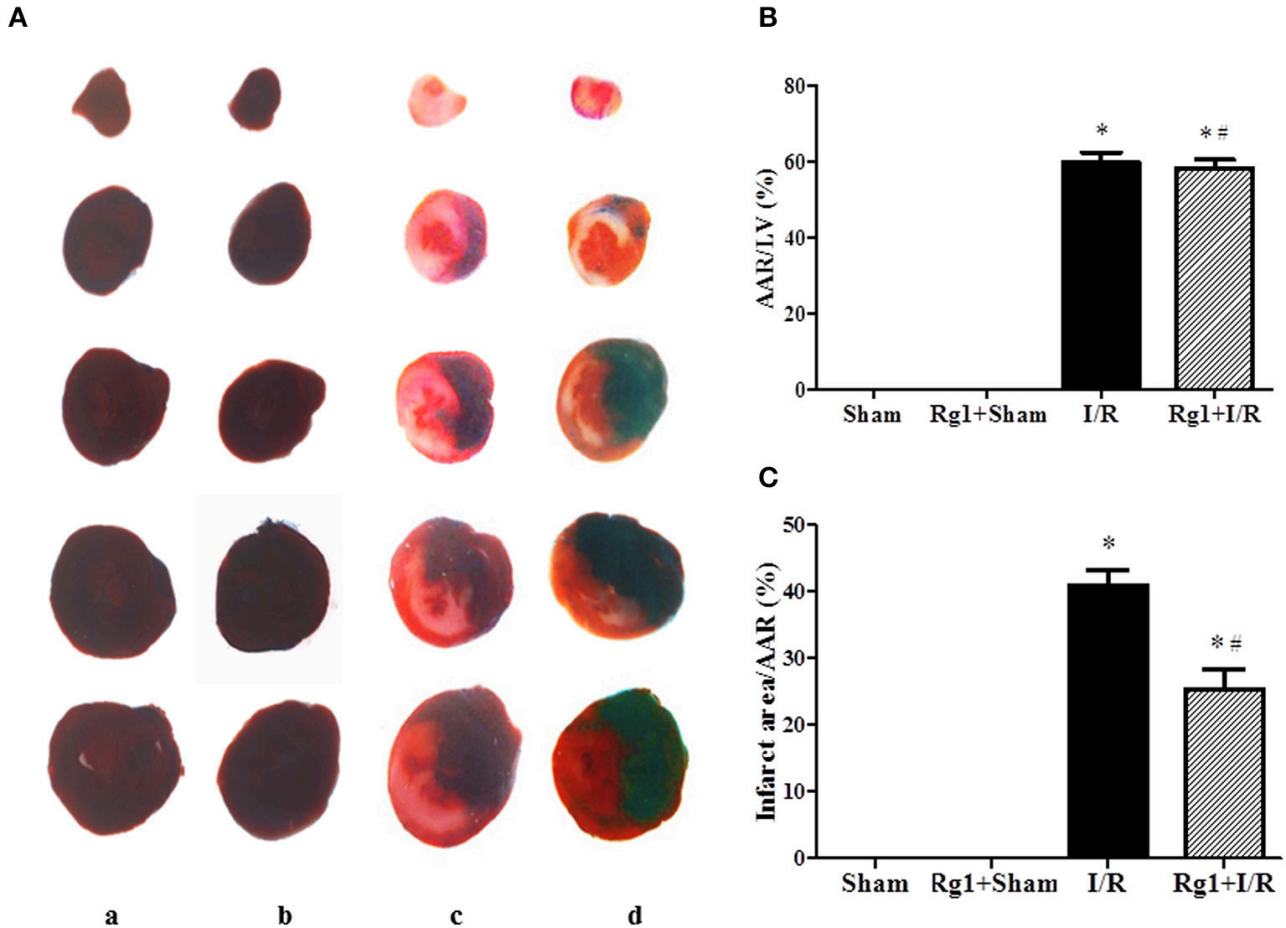
Effect of Rg1 on myocardial infarct size of rats subjected to 30 min ischemia followed by 90 min reperfusion. **(A)** Representative images of myocardial tissues from different groups. The sections were stained with TTC and Evans blue at 90 min after I/R. **(B,C)** Quantitative evaluation of AAR/LV and infarct area/AAR area at 90 min after I/R. Sham, Sham group; Rg1+Sham, pretreatment with Rg1 alone group; I/R, I/R group; Rg1+I/R, pretreatment with Rg1 plus I/R group. Data are expressed as mean ± SE. ^*^p < 0.05 vs. Sham group. ^#^*p* < 0.05 vs. I/R group. *N* = 6.

We first examined whether Rg1 exhibited a cardioprotective role during I/R in rats. Myocardial infarct was assessed 90 min after I/R by Evans blue-TTC double staining, and the representative heart slices in different groups are shown in Figure 1A. Compared with Sham (a) and Rg1+ Sham (b) group, noticeable ischemia and infarct areas were observed in myocardial tissue slices in I/R group (c). Of notice, heart slices from Rg1+I/R groups (d) had an obviously smaller area of myocardial infarct while a similar area of ischemic region as compared with I/R group. The quantitative analysis of AAR/LV and infarct area/AAR depicted in Figures 1B,C showed a significant increase in AAR/LV and infarct area/AAR in I/R group compared with Sham group. Rg1 treatment did not affect AAR/LV but prevented the increase in infarct area/AAR significantly. This protective effect of Rg1 was not increased at higher concentration (10 mg/kg, Supplementary Figure 1).

### Rg1 recovers MBF during reperfusion

**Figure 2:**
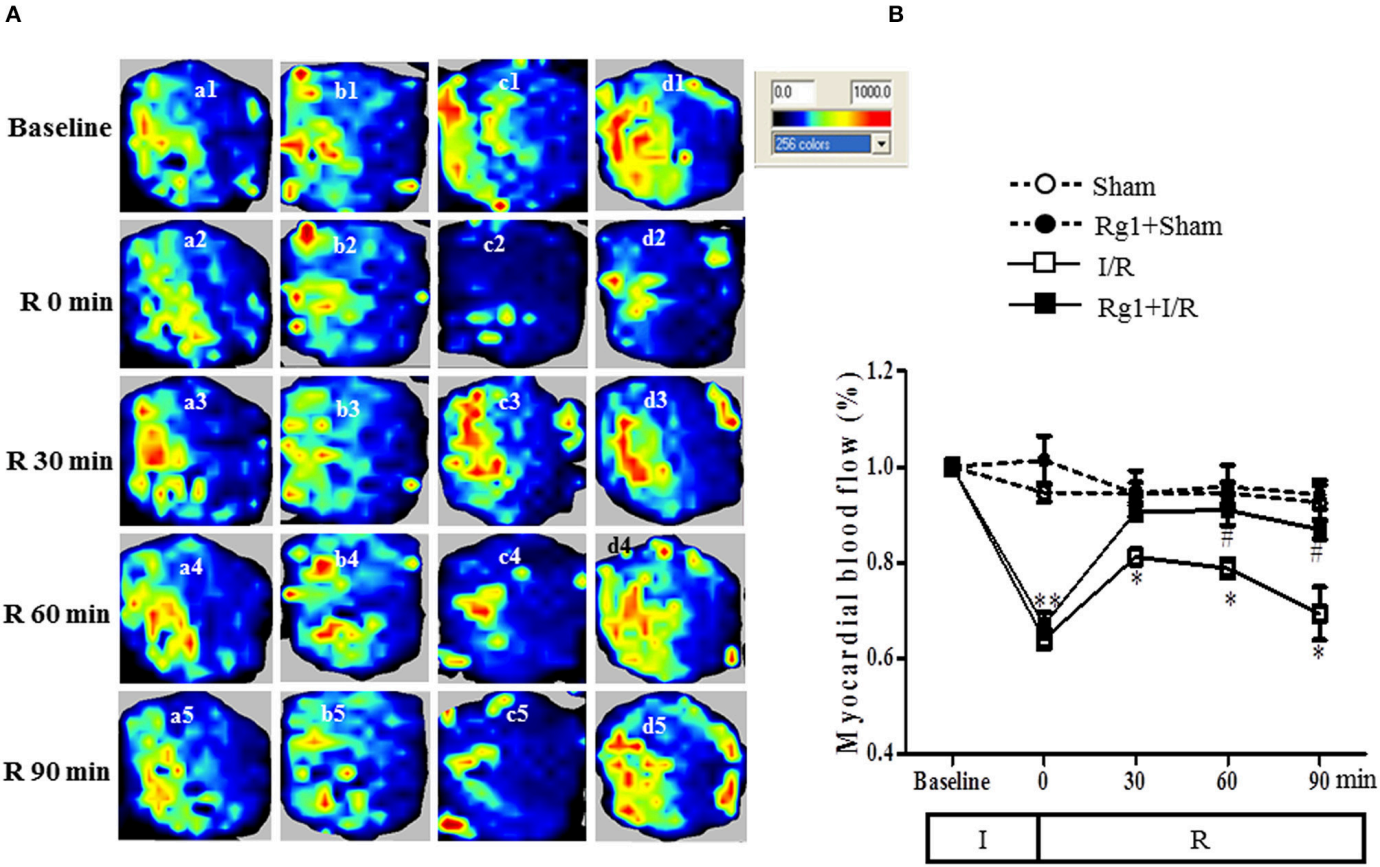
Effect of Rg1 on MBF. **(A)** Representative laser Doppler images of MBF in Sham (a), Rg1+Sham (b), I/R (c), and Rg1+I/R (d) group, respectively. 1, 2, 3, 4, 5 represents images acquired at baseline and 0, 30, 60, 90 min after I/R, respectively. **(B)** Time course of MBF of rats subjected to I/R in various groups. Sham, Sham group; Rg1+Sham, pretreatment with Rg1 alone group; I/R, I/R group; Rg1+I/R, pretreatment with Rg1 plus I/R group. Data are expressed as mean ± SE. ^*^p < 0.05, ^**^p < 0.01 vs. Sham group. ^#^*p* < 0.05 vs. I/R group. *N* = 6.

We then tested whether Rg1 might influence MBF during I/R. MBF was assessed by the Laser Scanning Doppler at different time points, and the representative images are presented in Figure 2A. No obvious difference in MBF at baseline was observed among the four groups (a1, b1, c1, and d1), and no changes were found in MBF in Sham and Rg1+Sham group at each time point tested, either. As expected, however, ischemia gave rise to an apparent decrease in MBF (c2 and d2) compared to the sham group (a2), which was recovered to a greater extent during reperfusion in Rg1+I/R group (d3, d4, and d5) than in the I/R group (c3, c4, and c5).

Figure 2B is the time courses of MBF changes in the four groups. As noticed, MBF in sham groups kept nearly constant over the entire observation. Ischemia caused a decrease in MBF in both I/R group and Rg1+I/R group to a level close to 60% of baseline. MBF in I/R group recovered to 80% of baseline at 30 min after reperfusion but gradually reduced to 70% of baseline 90 min after I/R. Impressively, MBF in Rg1+I/R group reversed to around 90% of baseline immediately after reperfusion, which persisted till 90 min after I/R, being significantly higher than that of I/R group.

### Rg1 ameliorates cardiomyocyte injury induced by I/R

**Figure 3:**
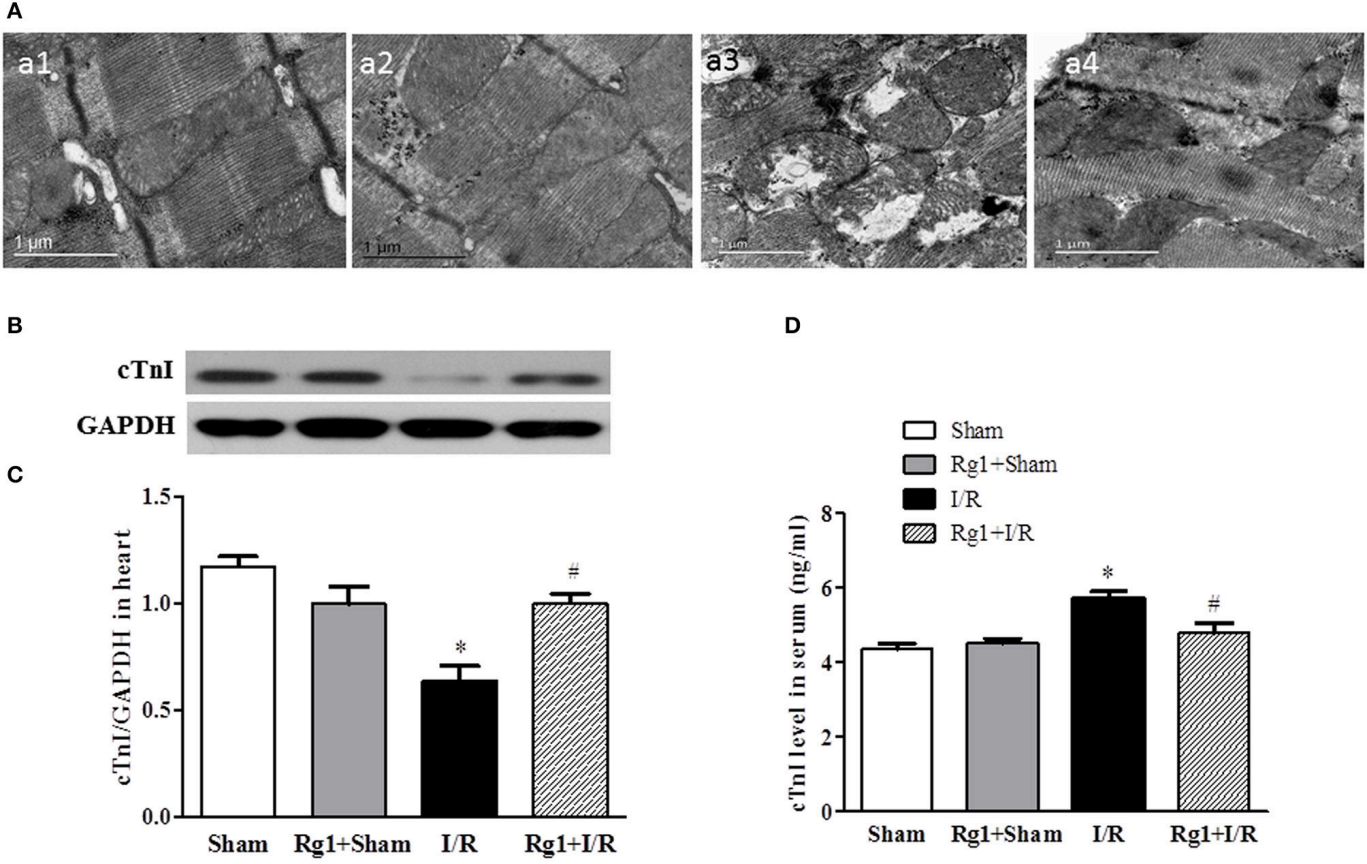
Effect of Rb1 on myocardial ultrastructure and cTnI levels in myocardial tissue and serum after I/R. **(A)** Presented are the representative electron micrographs of myocardium from different groups. **(B,C)** The representative western blotting images and semi-quantitative analysis of cTnI in myocardial tissue at 90 min after I/R. **(D)** The quantitative analysis of cTnI level in serum. Sham, Sham group; Rg1+Sham, pretreatment with Rg1 alone group; I/R, I/R group; Rg1+I/R, pretreatment with Rg1 plus I/R group. Data are expressed as mean±SE. ^*^p < 0.05 vs. Sham group. ^#^*p* < 0.05 vs. I/R group. *N* = 6.

The structure of cardiomyocytes was examined by electron microscopy in different groups, the results are displayed in Figure 3A. Obviously, the myocardium in Sham (a1) and Rg1+sham (a2) group revealed a typical normal ultrastructure, such as the well-preserved mitochondria with evenly distributed dense matrix, and regularly arranged myofibrils. By contrast, the myocardium ultrastructure in I/R (a3) group exhibited apparent injury, manifested swelling mitochondria and disrupted myofibrils, which were, however, ameliorated by Rg1(a4).

The potential of Rg1 to protect against myocardial I/R injury was further verified by test of the cardiac troponin I (cTnI) in myocardium and blood plasma, using western blot (Figures 3B,C) and ELISA (Figure 3D), respectively. In support of the results of electron microscopy, the levels of cTnI decreased in myocardium and increased in blood plasma in response to I/R challenge. The change in cTnI level in both myocardium and blood plasma elicited by I/R was significantly protected by Rg1 treatment.

### Rg1 improves heart function during I/R

**Figure 4:**
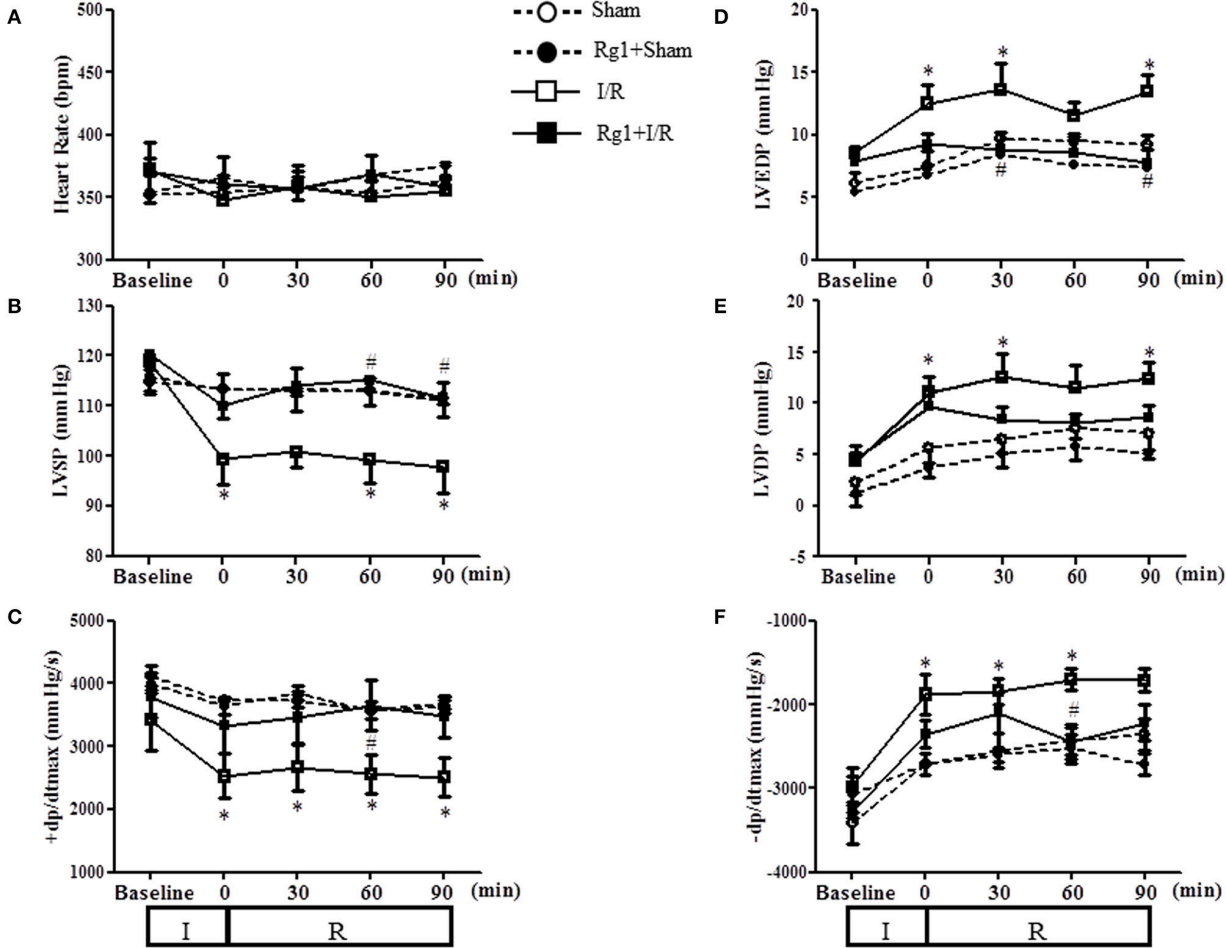
Effect of Rg1 on cardiac function. Presented are the time courses of HR **(A)**, LVSP **(B)**, +dp/dtmax **(C)**, LVEDP **(D)**, LVDP **(E)**, and −dp/dtmax **(F)** in Sham, Rg1+Sham, I/R and Rg1+I/R group, respectively, at baseline and 0, 30, 60, 90 min after I/R. Sham, Sham group; Rg1+Sham, pretreatment with Rg1 alone group; I/R, I/R group; Rg1+I/R, pretreatment with Rg1 plus I/R group. Data are expressed as mean ± SE. ^*^p < 0.05 vs. Sham group. ^#^*p* < 0.05 vs. I/R group. N = 6.

We next assessed the effect of Rg1 on the left ventricular function during I/R challenge. As illustrated in Figure 4, in comparison with Sham group, ischemia caused a significant decline in LVSP and +dp/dtmax, and an increase in LVDP, LVEDP and −dp/dtmax, which persisted over the observation, indicating an impairment of heart function that initiated during ischemia and did not recovered upon reperfusion. Evidently, all the impairments but LVDP were protected from by pre-treatment with Rg1.

### Rg1 inhibits myocardial apoptosis induced by I/R

**Figure 5:**
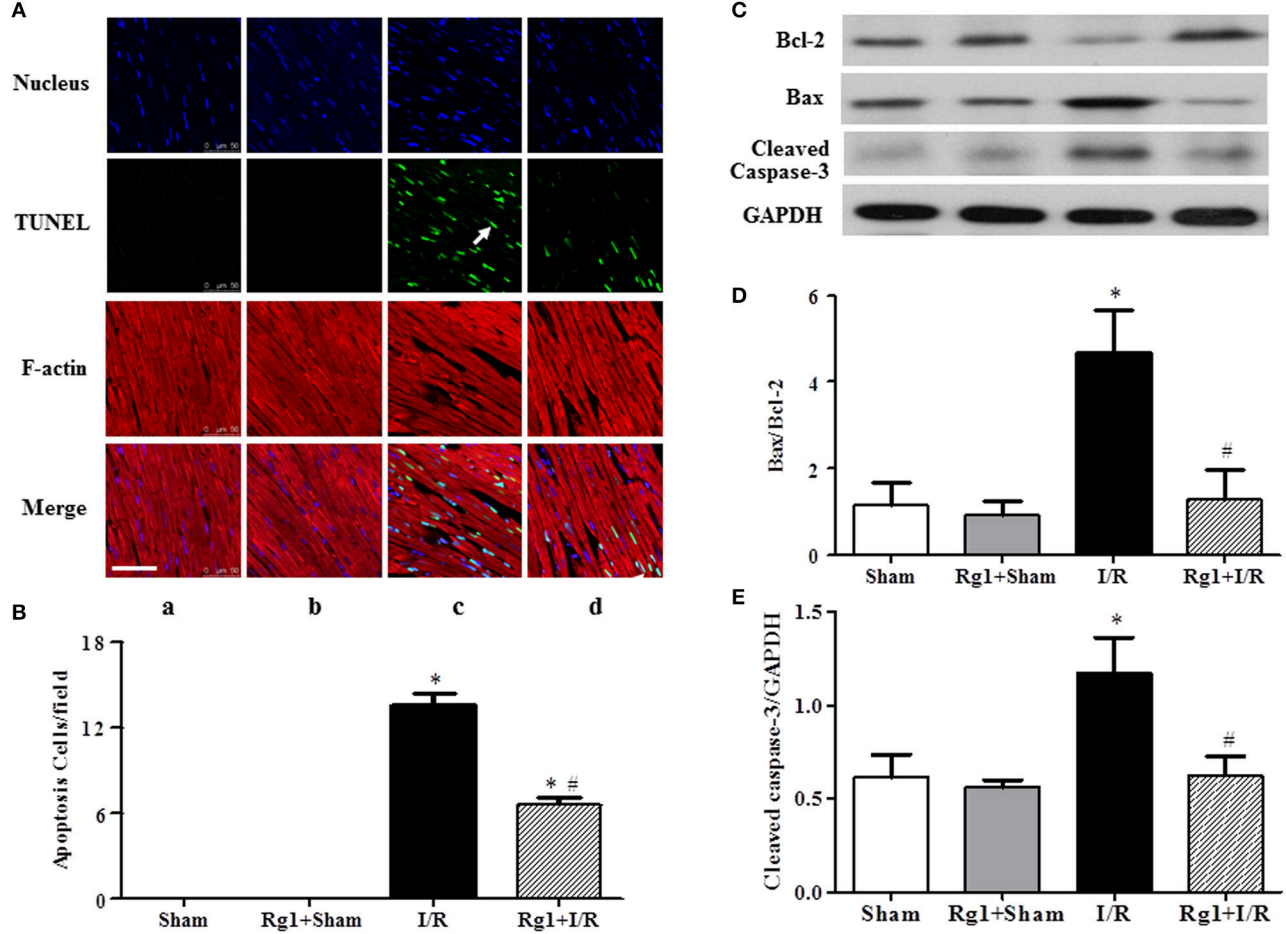
Effect of Rg1 on myocardial apoptosis and F-actin. **(A)** Presented are the representative photographs of double staining of F-actin and TUNEL. Nuclei are stained with blue, F-actin red, and TUNEL-positive cells green (arrow). Bar = 50 μm. **(B)** Quantitative analysis of TUNEL positive cells among the various groups. The expression of Bcl-2, Bax, and cleaved-caspase-3 in the myocardial tissue from different groups was detected by western blotting with the representative western blotting image of each group showing in **(C)**, semi-quantification of Bax/Bcl-2 ratio in **(D)** and cleaved-caspase-3 in **(E)**, respectively. Sham, Sham group; Rg1+Sham, pretreatment with Rg1 alone group; I/R, I/R group; Rg1+I/R, pretreatment with Rg1 plus I/R group. Data are expressed as mean±SE. ^*^p < 0.05 vs. Sham group. ^#^*p* < 0.05 vs. I/R group. *N* = 6.

To further investigate the protective role of Rg1 in the myocardium subjected to I/R stimulation, double staining with rhodamine phalloidin and TUNEL was performed for the surrounding of infarction areas of the LV myocardial tissue, with the representative images from different groups illustrated in Figure 5A, wherein cardiomyocyte thin filaments F-actins are labeled by rhodamine phalloidin as red, while apoptotic cardiomyocyte nuclei stained by TUNEL as green. Noticeably, I/R caused degradation of F-actin and rupture of myocardial fibers in I/R group (c), as well as cardiomyocyte apoptosis, which were alleviated by Rg1 with significant deduction in apoptotic cells (d) (Figure 5B). This result validated the potential of Rg1 to protect cardiomyocyte from I/R injury, and suggested the involvement of apoptosis in its underlying mechanism. In line with this result, western blot showed a significant increase in the ratio of Bax/Bcl-2 (Figures 5C,D) and the expression of cleaved-caspase-3 (Figures 5C,E) in myocardial tissue in I/R group, and such alterations were suppressed by Rg1.

### Rg1 regulates energy metabolism-related proteins

**Figure 6:**
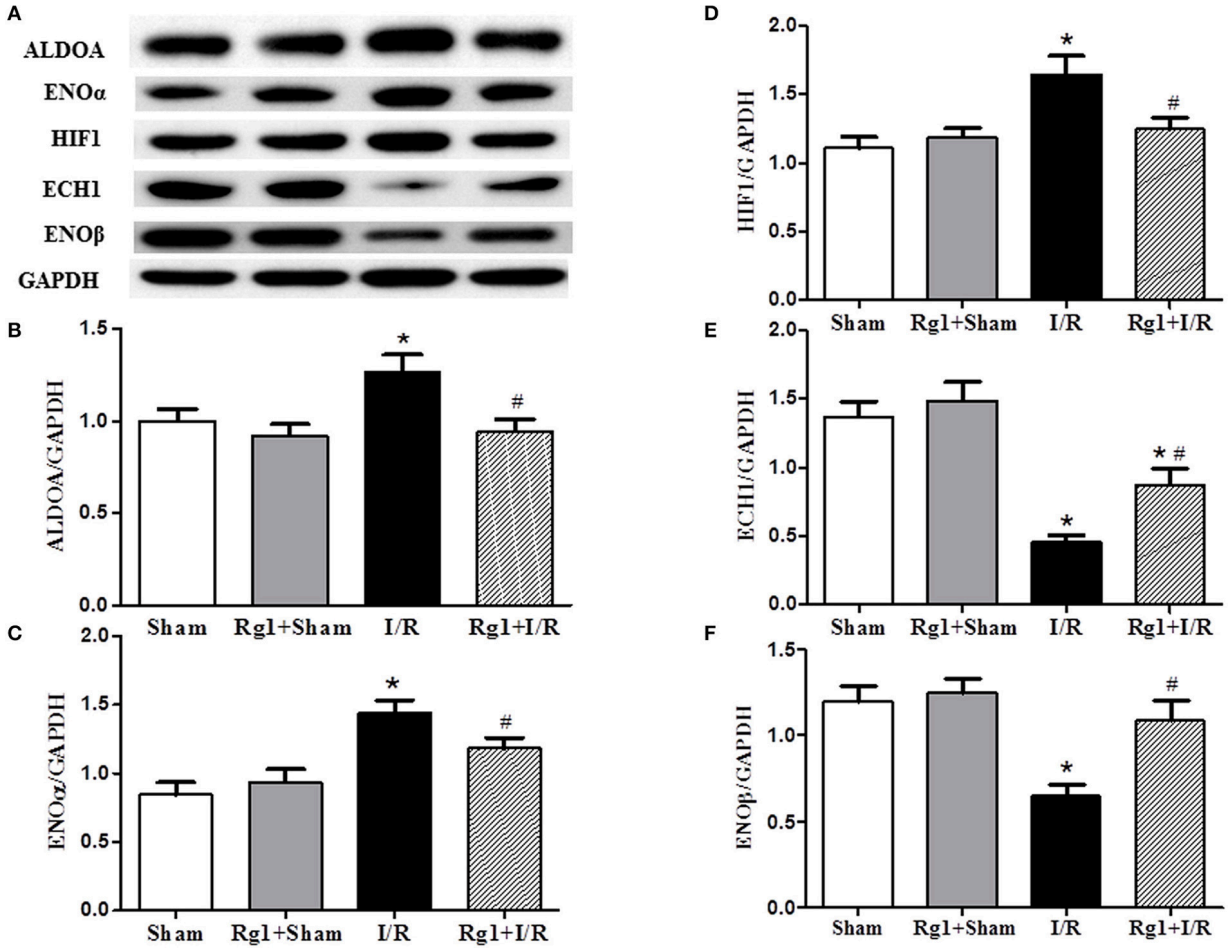
Effect of Rg1 on the expression of energy metabolism related proteins in the myocardial tissue. The representative western blotting image of each protein in each group is displayed in **(A)**, with semi-quantitative analysis of ALDOA, ENOα, HIF1, ECH1, and ENOβ shown in **(B–F)**, respectively, which were detected in myocardial tissue at 90 min after I/R. Sham, Sham group; Rg1+Sham, pretreatment with Rg1 alone group; I/R, I/R group; Rg1+I/R, pretreatment with Rg1 plus I/R group. Data are expressed as mean ± SE. ^*^p < 0.05 vs. Sham group. ^#^*p* < 0.05 vs. I/R group. *N* = 6.

Cardiac I/R is known to cause an increase in glycolysis and a decrease in fatty acid oxidation, we thus detected the expression of proteins related to fatty acid oxidation and glycolysis. Western blot showed that HIF1, ENOα, and ALDOA were increased (Figures 6A–D), while ECH1 and ENOβ (Figures 6A,E,F) were decreased in myocardial tissue in I/R group compared with Sham and Sham+Rg1 groups. All the changes of the proteins were ameliorated by Rg1, suggesting the beneficial role of Rg1 in normalizing energy metabolism (Figures 6A–F).

### Rg1 prevents against the impairment in respiratory chain complexes activity and ATP deprivation after I/R

**Figure 7:**
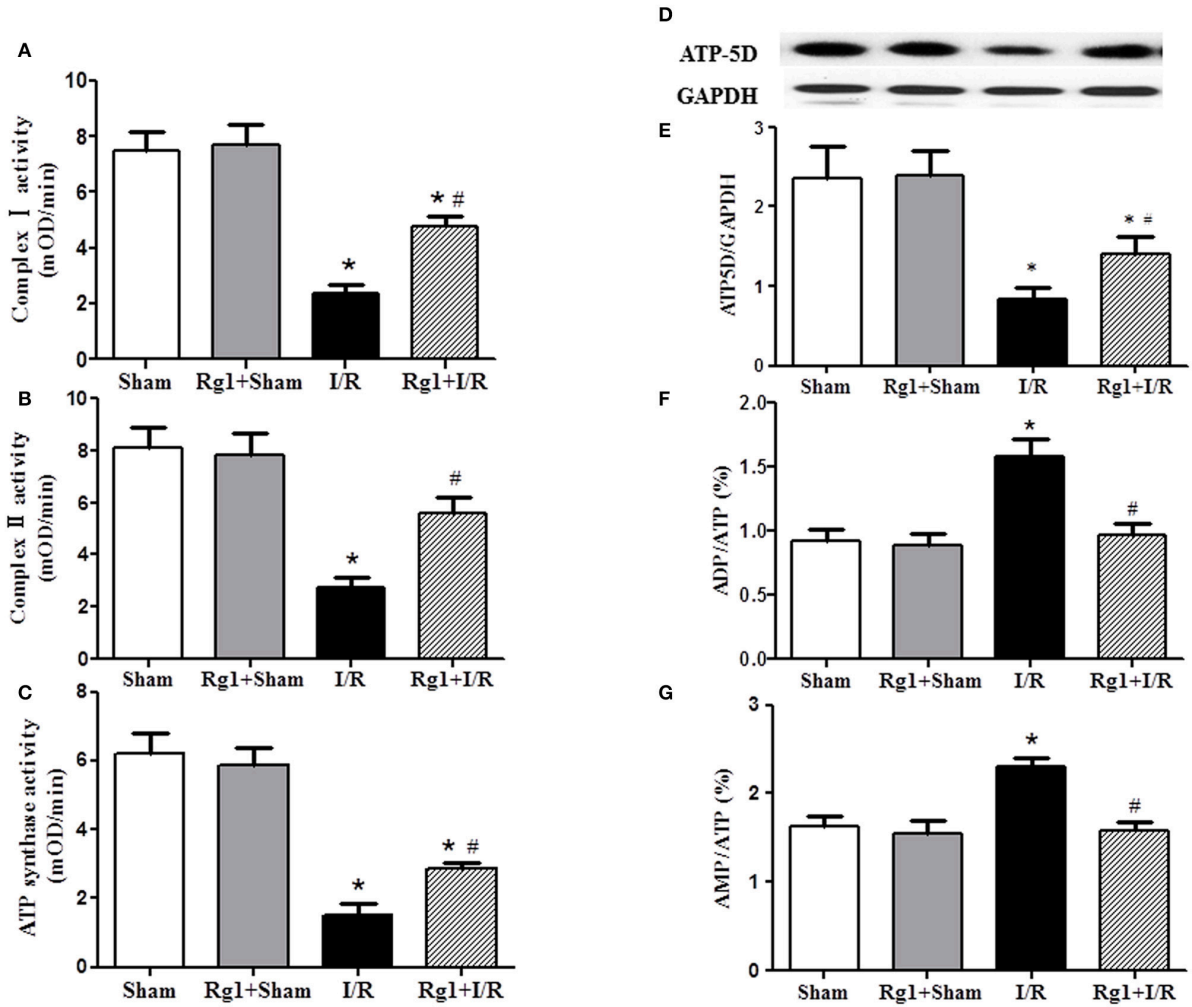
Effect of Rg1 on the activity of mitochondria respiratory chain Complexes and the expression of ATP5D in the myocardial tissue of rats subjected to I/R. **(A–C)** The effect of Rg1 on the activity of complex I, complex II and ATP synthase, respectively, in myocardial tissue at 90 min after I/R. **(D,E)** The representative western blotting images and semi-quantitative analysis of ATP5D in myocardial tissue at 90 min after I/R. **(F**,**G)** Effects of Rg1 on the ratio of ADP/ATP, and AMP/ATP in myocardial tissue at 90 min after I/R. Sham, Sham group; Rg1+Sham, pretreatment with Rg1 alone group; I/R, I/R group; Rg1+I/R, pretreatment with Rg1 plus I/R group. Data are expressed as mean ± SE. ^*^p < 0.05 vs. Sham group. ^#^*p* < 0.05 vs. I/R group. *N* = 6.

The activity of respiratory chain complex I, II, and ATP synthase was investigated, which are responsible for the ATP output and oxygen free radicals production from respiratory chain. Figures 7A–C showed that the activity of complex I, complex II, and ATP synthase was decreased significantly in myocardial tissue after I/R compared with that in Sham and Rg1+Sham groups, while Rg1 administration ameliorated the dysfunction of the complex I, complex II, and ATP synthase significantly.

To gain insight the rationale behind the alteration of ATP synthase in different groups, western blotting was undertaken to detect the expression of ATP synthase δ-subunits ATP5D. The result revealed a decreased expression of ATP5D in I/R group, and Rg1 reversed this change (Figures 7D,E). In agreement with the results above, ADP/ATP and AMP/ATP ratio increased dramatically 90 min after I/R (Figures 7F,G), indicating a dysregulation in the balance of energy metabolism moving toward ATP catabolism. However, Rg1 significantly prevented ATP from degradation after I/R.

### Rg1 attenuates RhoA/ROCK activation induced by I/R

**Figure 8:**
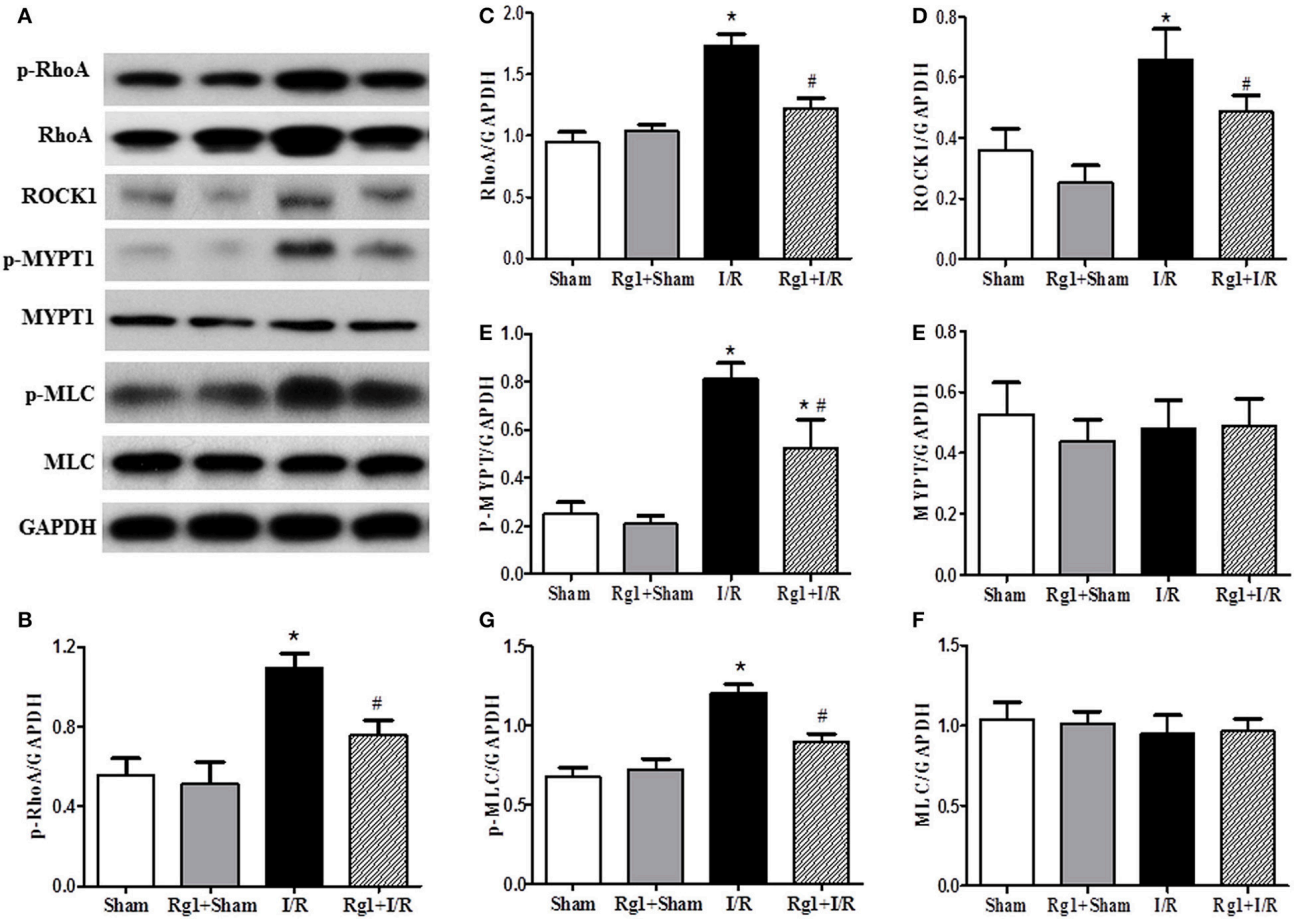
Effect of Rg1 on the expression and phosphorylation of RhoA/ROCK signaling pathway in the myocardial tissue of rats subjected to I/R. **(A)** Representative western blotting images of RhoA/ROCK signaling pathway related proteins in the myocardial tissue. **(B–H)** Semi-quantitative analysis of p-RhoA, RhoA, ROCK1, p-MYPT1, MYPT1, p-MLC, MLC, respectively, in myocardial tissue at 90 min after I/R. Sham, Sham group; Rg1+Sham, pretreatment with Rg1 alone group; I/R, I/R group; Rg1+I/R, pretreatment with Rg1 plus I/R group. Data are expressed as mean ± SE. ^*^p < 0.05 vs. Sham group. ^#^*p* < 0.05 vs. I/R group. *N* = 6.

By regulating the assembly and organization of actin cytoskeleton, RhoA/ROCK signaling pathway is capable of controlling the proteins expression that are responsible for modulating the cardiovascular system (Cai et al., 2016). We thus assessed the expression of proteins implicated in RhoA/ROCK signaling pathway by using western blotting. The results showed a significant increase in the phosphorylation and expression of RhoA, and the expression of ROCK1 in I/R-elicited myocardial tissue compared with Sham and Rg1+Sham groups, which was noticeably inhibited by Rg1 treatment (Figures 8A–D).

As two important effectors of ROCK1, the expression of MYPT-1 and MLC was also evaluated by western blotting. The results indicated that myocardial I/R resulted in an increase of phosphorylation of MYPT-1 and MLC compared with Sham and Rg1+Sham group. Rg1 administration significantly attenuated the phosphorylation level of MYPT-1 and MLC after I/R (Figures 8A,E–H).

### Rg1 is able to bind to RhoA in a saturable manner

**Figure 9:**
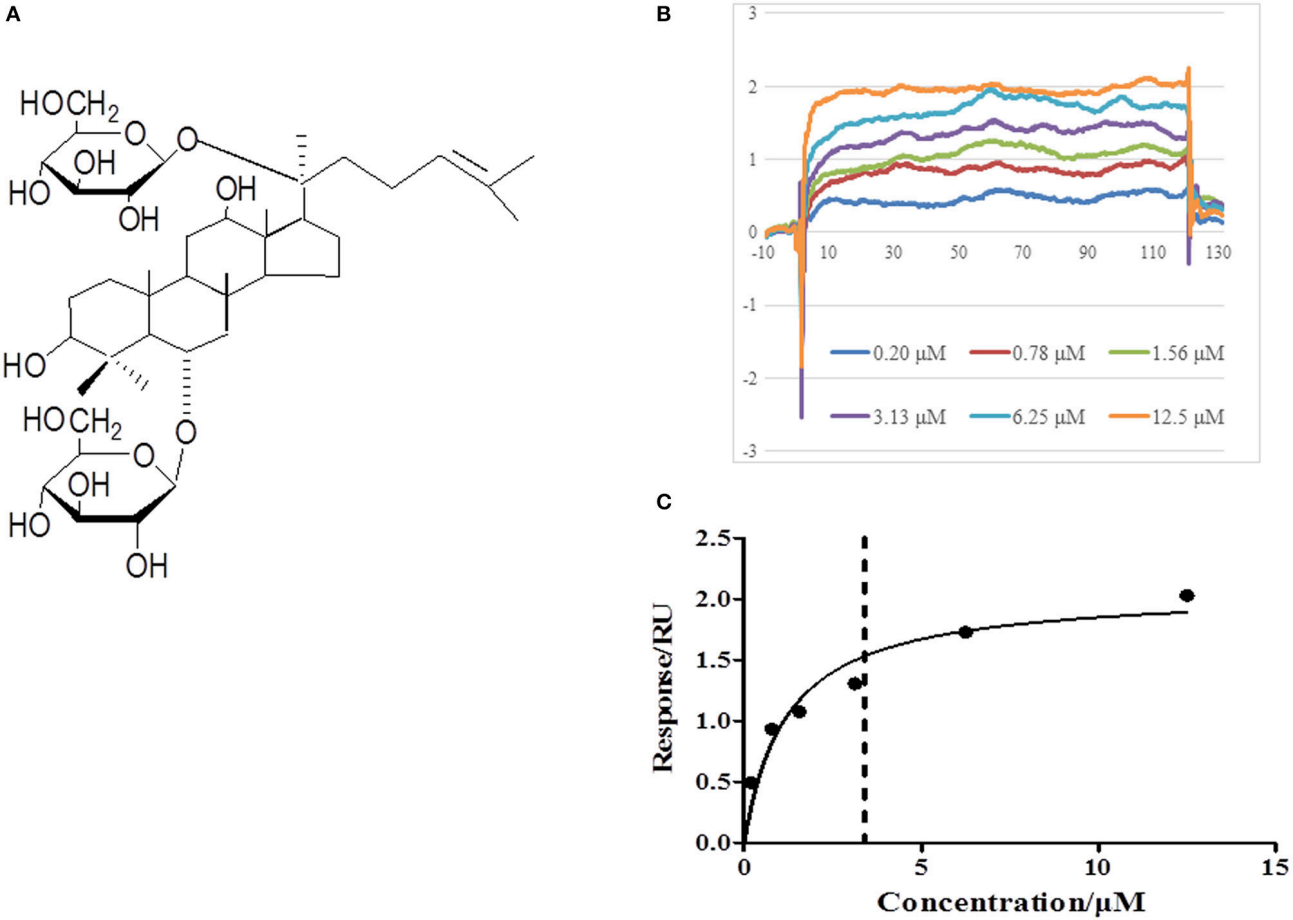
Rg1 binds to RhoA. **(A)** The chemical structure of Rg1. **(B,C)** The affinity of Rg1 to RhoA tested by SPR. Shown are the representative sensorgrams obtained from the injections of Rg1 at different concentrations using SPR.

In view of the central role of RhoA/ROCK signaling pathway in transcription of diverse proteins, we next explored the potential of Rg1 to bind to RhoA by using SPR. As shown in Figures 9B,C, Rg1 bound to RhoA in a saturable manner, indicating a specific binding between RhoA and Rg1. The equilibrium dissociation constant (KD) for Rg1 bound to RhoA was 3.403 × 10^−6^ (M).

## Discussion

The present study revealed that pretreatment with Rg1 protected heart from I/R-induced myocardial impairment, as demonstrated by the decrease of myocardial infarction size, the increase in MBF, and ameliorating of cardiomyocyte structure and function, consistent with previous reports (Li et al., 2017), which provides further support for the feasibility to apply Rg1 in clinic for the patients vulnerable to myocardial I/R. Importantly, the evidence from the present study highly suggests that the cardioprotective effect of Rg1 is mediated by a mechanism involving modulation of energy metabolism enhancing activity and expression of mitochondria respiratory chain Complexes via RhoA/ROCK signaling pathway.

Myocardial I/R injury manifests multiple insults which result from the interaction of a spectrum of impairments. As expected, the present study found that MBF was decreased by ischemia manipulation, which recovered to some extent upon reperfusion, though, but never reached to that of sham group over the time of observation. This result represents a phenomenon so called no reflow, which is believed to arise from the disturbed microcirculation, such as hyperpermeability of microvascular endothelium. Deprivation of energy during I/R impairs microvascular endothelium barrier, leading to leakage of plasma albumin and interstice edema, which imposes a pressure on the microvessels thus hinders complete recovery of MBF upon reperfusion. Rg1 pretreatment abolished the no reflow phenomenon, which is likely attributable to its potential to attenuate the dysfunction of energy metabolism during I/R. More interestingly, Rg1 pretreatment improved heart function even before the recovery of MBF, implying that Rg1 protected cardiomyocyte contraction strength from decline in the ischemia phase. Theoretically, the improved myocardial flow may increase the supply of oxygen thus attenuate energy metabolism which helps limit the I/R injury; the limited I/R injury in, e.g., microcirculation and cardial myocyte contraction may in return improve the myorcardial flow, forming a positive feedback to drive the protecting process of the drug. On the other hand, we always keep it in mind that the improved myocardial flow is a double side sword for I/R injury, which may also result in a burst of ROS that exacerbates the ischemic injury. The effect of improved myocardial flow on I/R injury seems to depend on the context concerned.

The role Rg1 played in this circumstance was likely accomplished by improvement of energy metabolism, given the fact that cardiomyocyte contraction strength is tightly controlled by the availability of ATP, which provides the energy for the sliding between thin and thick filament and is essential for keeping the integrity of F-actin as well (Korn et al., 1987; Han and Ogut, 2010).

In addition to dysregulation of cardiac function, I/R resulted in cardiomyocyte damage, as observed by morphological evaluation in the present study. Both necrosis and apoptosis are known to contribute to the I/R-elicited myocardium injury with the first type of cell death being dominant in ischemia phase while the second in the reperfusion phase (Han et al., 2017). Noticeably, both types of cell death are likely associated with disturbed energy metabolism. In ischemia, hypoxia and lack of glucose result in deprivation of ATP that causes failure of ion pumps in plasma membrane and increase in osmotic pressure of cytoplasm, eventually leading to cell collapse and necrosis (Kagawa, 1984; Ong and Gustafsson, 2012). On the other hand, as the major source of ATP in physiological condition, mitochondria are well recognized to mediate the progress of apoptosis in response to stress. Obviously, normally functioning mitochondria with sufficient production of ATP are required for fighting against I/R injury. The present study demonstrated the ability of Rg1 to attenuate myocardial I/R injury, in line with the reports from others, suggesting that Rg1 may target some signaling that regulates mitochondria function. Taken together, the results from functional and morphological assessments have brought energy metabolism into our sight field as a likely target for Rg1 to act.

To test this assumption, we first evaluated the energy metabolism status in different conditions, and found that the increased ratios ADP/ATP and AMP/ATP in I/R group were significantly protected by Rg1 pretreatment, indicating the efficiency of Rg1 in improving energy metabolism. Two mechanisms possibly exist thereby Rg1 improves energy metabolism. One is to enhance oxygen supply, so that increases the recipient of electron and the end product ATP. Given the potential of Rg1 to abolish the no reflow in the reperfusion phase, this mechanism is certainly a contributor in this respect, although the cause and effect relation between its effect on improving MBF and energy metabolism remains an open question. Another mechanism is to modulate the activity and/or expression of mitochondria respiratory chain Complexes, the machinery for synthesis of ATP (Consolini et al., 2017). The results from the present study demonstrated that this mechanism contributes indeed to the energy improving role of Rg1 in myocardial I/R injury, as evidenced by the increase in the activity of these Complexes by Rg1 pretreatment. Among the three respiratory chain complexes tested in the present study, Complex I and II are responsible for the transport of electrons, adequately working of which is essential not only for synthesis of ATP, and also for protecting the escape of electrons, a resource for generation of superoxide. Complex V, or ATP synthase, synthesizes ATP using the potential energy stored as a proton gradient across the inner membrane of mitochondria. Noticeably, Rg1 increased the protein expression of ATP5D, in addition to the activity of Complex V, implying that Rg1 possibly acts at somewhere upstream that regulate protein expression. This notion was supported by the examination of some other enzymes that altered expression in response to I/R injury (Lopaschuk and Stanley, 2006; Frank et al., 2012; Patkar et al., 2012), including ALDOA, ENOα, HIF1, ECH1, and ENOβ, the enzymes that participate in glycolysis or fatty acid oxidation (Keller et al., 1995; Mizukami et al., 2004; Lopaschuk and Stanley, 2006; Papandreou et al., 2006; Patkar et al., 2012), which were either upregulated or downregulated after I/R challenge. Of notice, all the I/R-induced changes in the expression of these proteins were significantly protected by Rg1, pointing again toward a target for Rg1 to act that regulates a broad spectrum of signaling pathways to modulate the expression of a diversity of proteins. RhoA/Rock is likely a candidate for such a target in view of its well-recognized potential in this regard.

Growing evidence indicates that RhoA and its main effector ROCK play a central role in a wide range of cardiovascular diseases such as congestive heart failure, atherosclerosis, and hypertension (Loirand et al., 2006; Hamid et al., 2007; Dong et al., 2010). A proteomic study has provided evidence that ROCK inhibitor increases the expression of lactate dehydrogenase and glyceraldehyde-3-phosphate dehydrogenase, as well as the level of ATP synthase α subunit during myocardial I/R injury (Cadete et al., 2010). Our previous study revealed that ROCK inhibitor pretreatment significantly increases the ATP content, ATP synthase activity and ATP5D expression in H/R-stimulated H9c2 cells (He et al., 2014), suggesting involvement of ROCK in the impaired energy production. In addition, inhibition of ROCK activity was found to reduce cardiomyocyte apoptosis through up-regulating Bcl-2 expression in myocardium (Bao et al., 2004; Surma et al., 2011). In agreement with these results, we found in the present study that Rg1 down-regulated the RhoA/ROCK signal pathway, manifested by the decrease in the phosphorylation of RhoA, MYPT-1, and MLC in myocardium, which was parallel with its attenuating effect on I/R-induced myocardial injury and energy metabolism disorder, highly suggesting RhoA/ROCK signal pathway as a central player in Rg1 action. This speculation was further sustained by the result from SPR showing that Rg1 was able to bind to RhoA in a saturable manner, suggesting a specific binding occurring between the two molecules.

**Figure 10:**
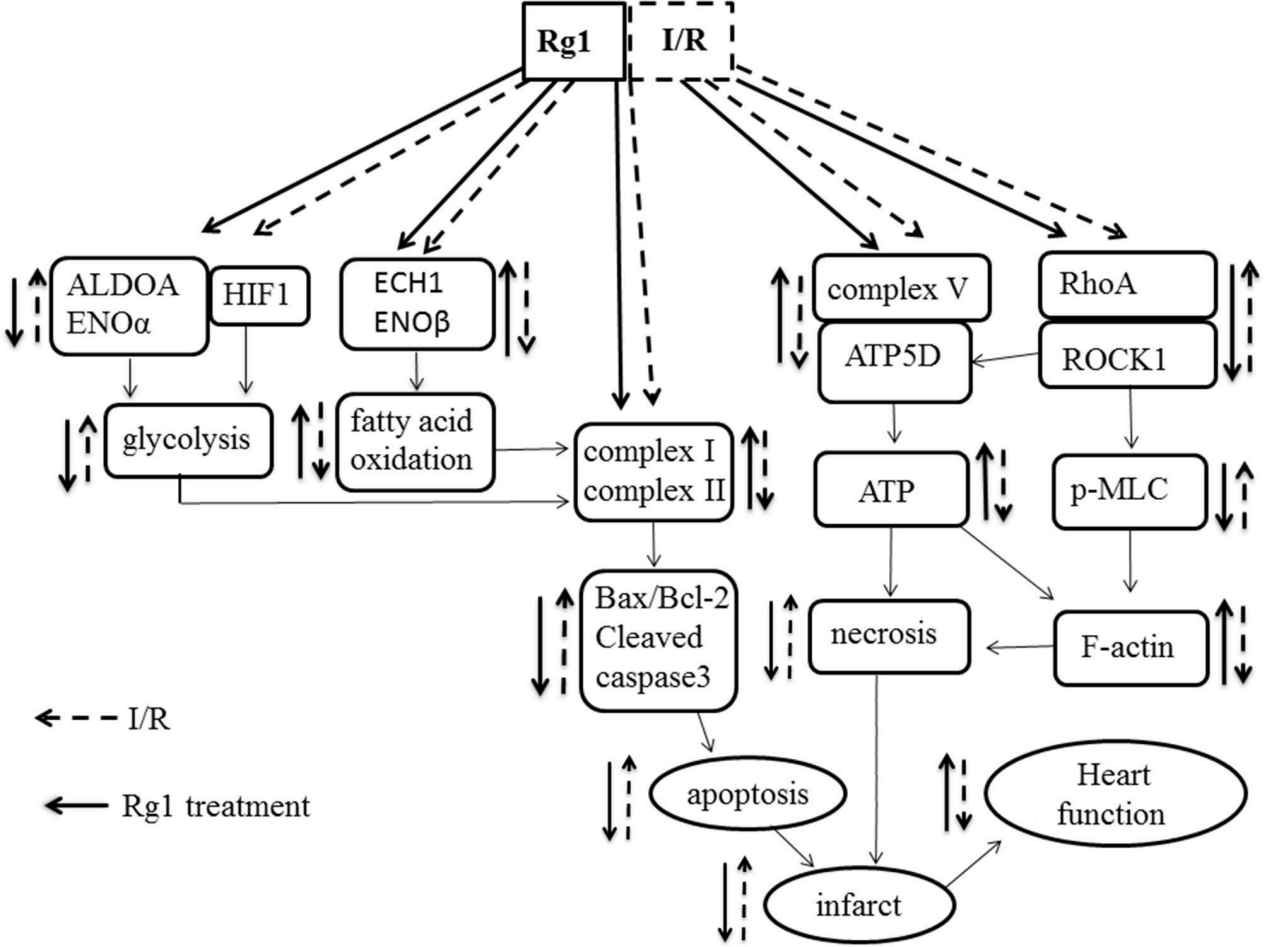
A diagrammatic sketch showing the pathways that lead to the various effects of Rg1 on protection of I/R-elicited myocardial injury by modulating energy metabolism. I/R induced the decrease of the expression of complex I, complex II, and ATP synthase in respiratory chain as well as the changes of enzymes involved in glycolysis and fatty acid oxidation, activated RhoA/ROCK1 modulating the expression of ATP5D in respiratory chain, which collectively result in myocardium infarction and heart dysfunction. Rg1 binds to RhoA and inactivates the RhoA / ROCK1 signaling, ameliorates the expression of complex I, complex II, and ATP synthase and enzymes involved in glycolysis and fatty acid oxidation, thus attenuates the I/R-induced insults.

In conclusion, pretreatment with Rg1 could protect myocardial structure and cardiac function from I/R injury. The cardiac protection afforded by Rg1 may attribute to its ability to adjust the energy metabolism via regulating the expression of energy metabolism related proteins, increasing the activity of mitochondria respiratory Complexes and the expression of ATP5D, all of which may at least partially be mediated through its binding to RhoA to inactivate the RhoA/ROCK pathway (Figure 10). These new findings provide evidence supporting Rg1 to be a novel option for protecting I/R-induced myocardial injury. Further studies are needed to address the detailed pathway(s) implicated in the benefit of Rg1.

## Author contributions

LL and C-SP: Performed the research, analyzed the data, and wrote the manuscript; LY, Y-YL, and KS: Contributed to animal experiments and immunochemistry analysis; H-NM, KH, and C-SP: Contributed to biochemical experiments; B-HH and XC: Contributed to electron microscopy analysis; Y-CC: Did the SPR experiment; J-YF and J-YH: Revised the manuscript; J-YH: Designed and funded the research; J-YH and LH: Interpreted the data and finally approved the submission of this manuscript. All authors have read and agreed with the manuscript.

### Conflict of interest statement

The authors declare that the research was conducted in the absence of any commercial or financial relationships that could be construed as a potential conflict of interest.

## Abbreviations

AAR: Area At Risk
ADP: Adenosine Diphosphate
AMP: Adenosine Monophosphate
AS-IV: Astragaloside IV
ATP: Adenosine Triphosphate
ATP5D: adenosine triphosphate synthase subunit delta
cTnI: Cardiac Troponin I
ELISA: Enzyme-linked Immunosorbent Assay
HE: Hematoxylin/eosin
I/R: Ischemia/Reperfusion
LA: Left Ventricular Area
PCI: percutaneous coronary intervention
Rg1: Ginsenoside Rg1
ROCK: Rho-associated Coiled-coil Containing Protein Kinase
SPR: Surface Plasmon Resonance.
