# Supplementary material for: Ginsenoside Rg1 Ameliorates Rat Myocardial Ischemia-Reperfusion Injury by Modulating Energy Metabolism Pathways
Source: Front Physiol. 2018 Feb 7;9:78. doi: 10.3389/fphys.2018.00078 (PMC5808323; doi:10.3389/fphys.2018.00078)
Supplement: Supplementary file 1 [file Image1.pdf]

Ginsenoside Rg1 ameliorates rat myocardial ischemia-reperfusion injury by modulating energy metabolism pathways

***Lin Li* [1,3,*], *Chun-Shui Pan* [3,4,5,6,7,*], *Li Yan* [3,4,5,6,7], *Yuan-Chen Cui* [3,4,5,6,7], *Yu-Ying Liu* [3,4,5,6,7], *Hong-Na Mu* [3,4,5,6,7], *Ke He* [3,4,5,6,7], *Bai-He Hu* [3,4,5,6,7], *Xin Chang* [3,4,5,6,7], *Kai Sun* [3,4,5,6,7], *Jing-Yu Fan* [3,4,5,6,7], *Li Huang* [1,†], and *Jing-Yan Han* [2,3,4,5,6,7,†]**

[1] Department of Integrative Cardiology, Beijing China-Japan Friendship Hospital, Beijing 100029, China.

[2] Department of Integration of Chinese and Western Medicine, School of Basic Medical Sciences, Peking University, Beijing 100191, China

[3] Tasly Microcirculation Research Center, Peking University Health Science Center, Beijing, 100191, China

[4] Key Laboratory of Microcirculation, State Administration of Traditional Chinese Medicine of the People's Republic of China, Beijing 100191, China

[5] Key Laboratory of Stasis and Phlegm, State Administration of Traditional Chinese Medicine of the People's Republic of China, Beijing 100191, China.

[6] Beijing Microvascular Institute of Integration of Chinese and Western Medicine, Beijing 100191, China.

[7] State Key Laboratory of Core Technology in Innovative Chinese Medicine, Beijing 100191, China.

Supplementary Materials: Supplementary Images for Figures 1

S Figure 1

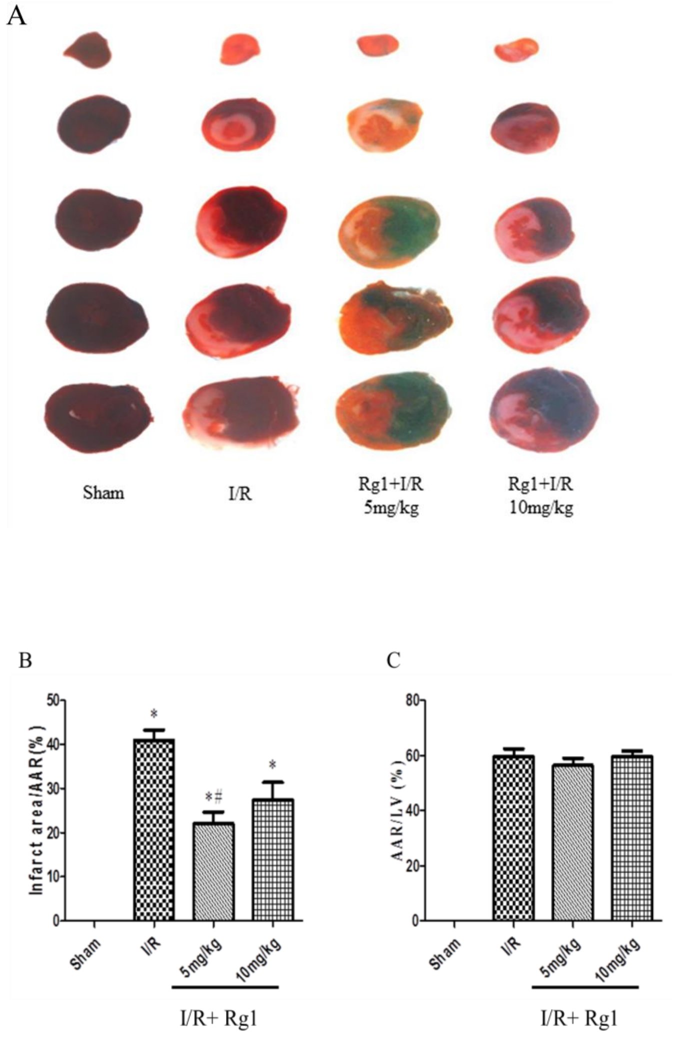

S Figure 1. Effect of Rg1 on myocardial infarct size of rats subjected to 30 min ischemia followed by 90 min reperfusion. (A) Representative images of myocardial tissues from different groups. The sections were stained with TTC and Evans blue at 90 min after I/R. (B) and (C) Quantitative evaluation of infarct area/AAR area and AAR/LV at 90 min after I/R. Sham, Sham group; I/R, I/R group; Rg1 5mg/kg+I/R, pretreatment with Rg1 5mg/kg plus I/R group; Rg1 10 mg/kg+I/R, pretreatment with

Rg1 10 mg/kg plus I/R group. Data are expressed as mean±SE. * $p < 0.05$ vs. Sham group. # $p < 0.05$ vs. I/R group. N=6.

Rg1 diminishes myocardial infarct size induced by I/R. Myocardial infarct was assessed 90 min after I/R by Evans blue-TTC double staining, and the representative heart slices in different groups are shown in Fig. 9A. Compared with Sham group, noticeable ischemia and infarct areas were observed in myocardial tissue slices in I/R group. Of notice, heart slices from Rg1+I/R (5mg/kg and 10mg/kg) groups had an obviously smaller area of myocardial infarct while a similar area of ischemic region as compared with I/R group. The quantitative analysis of infarct area/AAR depicted in Fig. 1B and 1C showed a significant increase in infarct area/AAR (B) and AAR/LV (C) in I/R group compared with Sham group. Both 5 mg/kg and 10 mg/kg Rg1 treatment did not affect AAR/LV but prevented the increase in infarct area/AAR significantly.
